# Molecular markers for early stratification of disease severity and progression in COVID-19

**DOI:** 10.1093/biomethods/bpac028

**Published:** 2022-11-02

**Authors:** Anusha Kashyap, Savitha Anne Sebastian, Sree Raksha Krishnaiyer NarayanaSwamy, KalyanKumar Raksha, Hanumanthappa Krishnamurthy, Bhuvana Krishna, George D’Souza, Jyothi Idiculla, Neha Vyas

**Affiliations:** Division of Molecular Medicine, St. John’s Research Institute, Bangalore, Karnataka 560034, India; Department of Medicine, St. John’s Medical College and Hospital, SJNAHS, Bangalore, Karnataka 560034, India; Department of Medicine, St. John’s Medical College and Hospital, SJNAHS, Bangalore, Karnataka 560034, India; National Centre for Biological Sciences, Bangalore Life Science Cluster, Bangalore, Karnataka 560065, India; National Centre for Biological Sciences, Bangalore Life Science Cluster, Bangalore, Karnataka 560065, India; Department of Critical Care Medicine, St. John’s Medical College and Hospital, SJNAHS, Bangalore, Karnataka 560034, India; Department of Pulmonary Medicine, St. John’s Medical College and Hospital, SJNAHS, Bangalore, Karnataka 560034, India; Department of Medicine, St. John’s Medical College and Hospital, SJNAHS, Bangalore, Karnataka 560034, India; Division of Molecular Medicine, St. John’s Research Institute, Bangalore, Karnataka 560034, India

**Keywords:** Cytokine-dependent, Cytokine-independent, COVID-19, SARS-CoV2, hypoxia, immunemodulator, IL6, IL8, biomarkers, decidualization

## Abstract

COVID-19 infections have imposed immense pressure on the healthcare system of most countries. While the initial studies have identified better therapeutic and diagnostic approaches, the disease severity is still assessed by close monitoring of symptoms by healthcare professionals due to the lack of biomarkers for disease stratification. In this study, we have probed the immune and molecular profiles of COVID-19 patients at 48-h intervals after hospitalization to identify early markers, if any, of disease progression and severity. Our study reveals that the molecular profiles of patients likely to enter the host-immune response-mediated moderate or severe disease progression are distinct even in the early phase of infection when severe symptoms are not yet apparent. Our data from 37 patients suggest that at hospitalization, interleukins (IL6) (>300 pg/ml) and IL8 levels (>200 pg/ml) identify cytokine-dependent disease progression. Monitoring their levels will facilitate timely intervention using available immunomodulators or precision medicines in those likely to progress due to cytokine storm and help improve outcomes. Additionally, it will also help identify cytokine-independent progressive patients, not likely to benefit from immunomodulators or precision drugs.

## Introduction

Coronaviruses are RNA viruses, which belong to the Coronaviridae family and subfamily Coronavirinae. Coronavirinae viruses are known to cause the common cold in humans. Bats or mice act as their natural host, and crossovers with humans are known since 1960 [1]. In 2002–2003, coronavirus designated as severe acute respiratory syndrome coronavirus (SARS-CoV) affected 8422 people in China and Hong Kong and caused 916 deaths (mortality rate of 11%) [2]. In 2012, the Middle East respiratory syndrome coronavirus affected 2494 people and caused 858 deaths (mortality rate of 34%) in Saudi Arabia [2]. In December 2019, the SARS-CoV2 virus was identified in Wuhan, China, and is responsible for the current pandemic, named COVID-19. SARS-CoV-2 is highly contagious and has spread to more than 180 countries. Mutations in the virus have improved its ability to cause human-to-human transmission [3, 4]. COVID-19 symptoms can range from flu-like symptoms to severe pneumonia, acute respiratory distress syndrome (ARDS), clotting disorder as well as multi-organ dysfunction syndrome (MODS), and death. COVID-19 has impacted more than 290 million people worldwide, and the numbers are constantly increasing. Healthcare resources and infrastructure have been stretched across the globe.

Clinically, COVID-19 infection triggers a biphasic illness. In the first phase, the virus infects the host and replicates causing flu-like symptoms, and most patients recover in 5–6 days without further complications. However, some patients enter the second phase characterized by hyper-inflammation causing pneumonia and/or clotting disorders. These complications are due to the virus-induced cytokine storm and cellular and organ damage. The virus-induced cytokine storm followed by hyper-inflammation causes severe pneumonia, ARDS, or MODS which can lead to death if not treated appropriately and timely. India is currently grappling with the third wave of COVID-19, and as of 4 January 2022, there are ∼35 million confirmed cases with around 1.3% deaths. The current therapeutic approach can cure many severe patients even with >50% lung damage if medical intervention is timely. While reliable tests are available for rapid and early detection of SARS-CoV-2 infection, there are no reliable tests available that can anticipate the second phase of the illness. Currently, this is addressed by close monitoring for onset of the symptoms like breathlessness and deterioration in oxygen saturation by which time it may already be too late to intervene. Here we attempted to identify early biomarkers for patients likely to enter the more serious second phase of the disease.

In the first part of the study, we evaluated the temporal profile of nine serum markers (cytokines and lung markers), lymphocytes, and neutrophils in 14 patients on the day of hospitalization (denoted as Day 0, i.e. Days 3–7 after the onset of symptoms) and at 48-h interval during their hospital stay, while the clinical course was also monitored. We found that the number of B- and T-cell subtypes shows variability with time but does not show a significant difference between mild nonprogressive and progressive patients. On the other hand, most cytokines and lung markers tested showed differential levels in mild versus severe disease. Based on this preliminary data, we selected five serum markers for analysis in a larger number of patients (37 patients) on Day 0.

Our study reveals that serum levels of interleukin (IL6), IL8, and surfactant protein-D (SP-D) can be used as early markers to stratify the severity of COVID-19, even on the day of hospitalization and before hypoxia sets in. IL6 and IL8 together can identify ∼50% of the patients with moderate or severe COVID-19 progression even when the severe symptoms of the disease progression are not yet apparent. This can aid in well-timed therapeutic intervention with available immunomodulators or precision medicine. Importantly, these markers can also identify patients who are progressive even in the absence of hyper-inflammatory response and are not likely to benefit from the immunomodulators or precision drugs like Tocilizumab.

## Results

### Neutrophil-to-lymphocyte ratio (NLR), T cells, or B cells are not different in progressive COVID-19 patients

**Figure 1:**
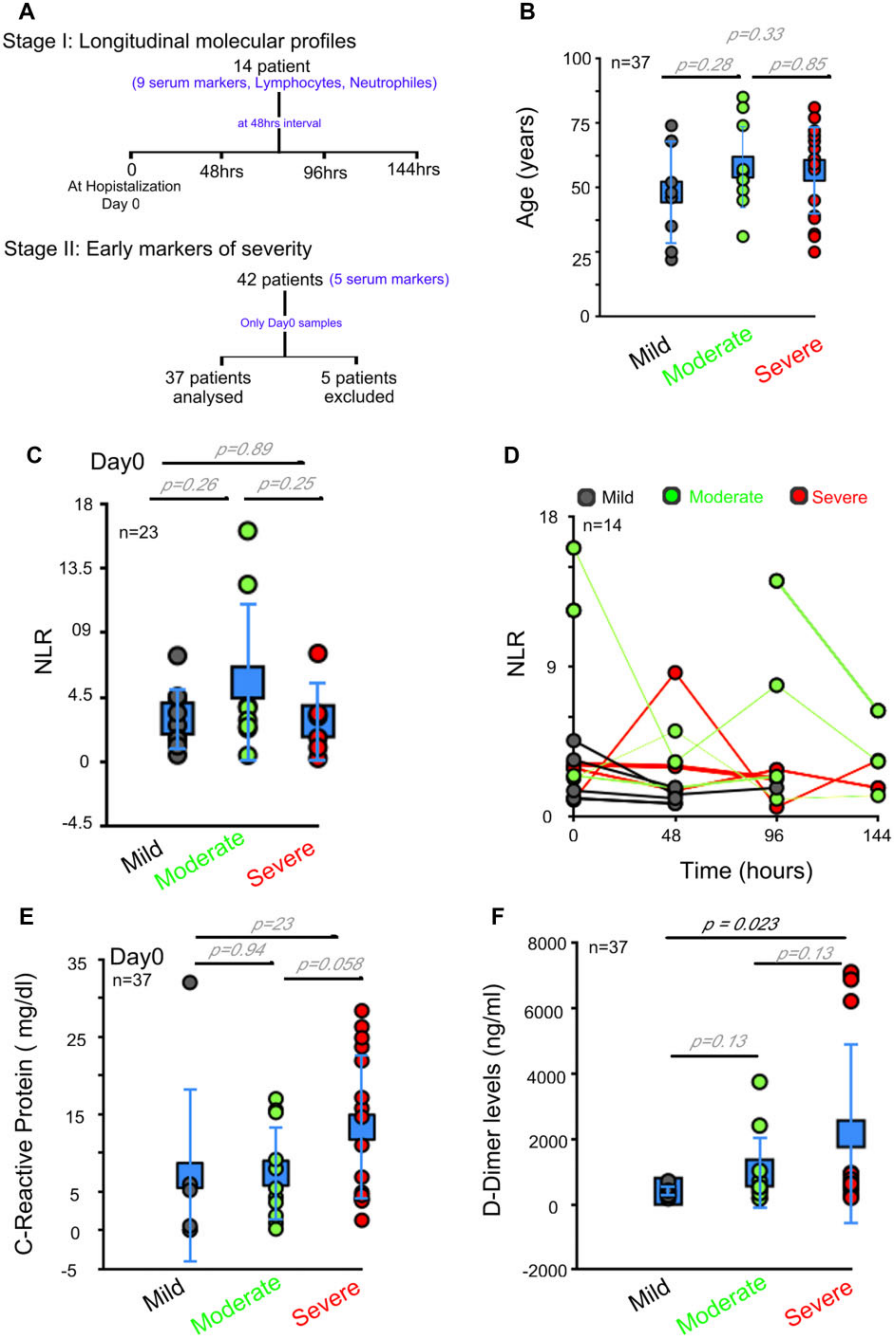
NLR and CRP fail to predict disease severity in COVID-19 patients. (**A**) Flow chart representing the study design. (**B**) Dot plot represents age of the patients included in the study. (**C**) Dot plot represents distribution of NLR in patients at the time of admission. (**D**) Line graph represents time-course analysis of NLR, at 48-h interval, FACS-based analysis. (**E**) Dot plot represents levels of CRP (mg/dl) among COVID-19 patients with varying severity on Day 0. (**F**) Dot plot represents levels of D-dimer among COVID-19 patients with varying severity. Gray, mild COVID-19; Green, moderate COVID-19; Red, severe COVID-19 patients. Day 0 is the hospital admission day. Error bars represents average ± SD across panels.

We recruited a total of 42 patients in this study, 5 patients with alcoholic liver disease or HIV were excluded from molecular analysis as these conditions are known to influence the molecular profiles of interest independently from SARS-CoV-2 infection or severity status [5, 6] (Fig. 1A and Supplementary Table S1). In the pilot stage of the study, venous blood and serum were collected from 14 patients for a time-course analysis of the molecular players of interest. This allowed us to map the temporal profile of the markers of choice and identify representative early molecular markers of progressive COVID-19. In the second stage of the study, only serum samples were collected on the day of admission (Day 0) to identify trends in a larger number of patients for necessary statistical significance (Fig. 1A). Molecular data were analyzed and correlated retrospectively with the clinical data. Clinically, patients are divided into three groups based on the percent saturation of the oxygen (SpO_2_): mild (SpO_2_ >94%), moderate (SpO_2_ 93–90%), or severe (SpO_2_ <90%) (Supplementary Fig. S1). The lowest SpO_2_ levels during the stay is being considered for the clinical classification.

We first analyzed the known markers for predicting the severity of the disease, which include neutrophil to lymphocyte ratio (NLR) [7–9], D-dimer [8–10], and C-reactive protein (CRP) levels [8]. The chronological age of patients in our study ranged between 20 and 80 years and disease severity was independent of the age (Fig. 1B and Supplementary Table S1). NLR was not different between mild, moderate, and severe disease at the time of admission (Fig. 1C) or during their hospitalization phase (Fig. 1D). CRP levels, a popular inflammatory marker, were also similar between mild (mean value 7.06 ± 11.32 mg/dl), moderate (7.36 ± 6.12 mg/dl), and severe disease (13.30 ± 9.43 mg/dl) for its reliable clinical use (Fig. 1E). D-dimer levels were different between the patients with mild (395.429 ± 179.24 ng/ml), moderate (954.81 ± 1111.24 ng/ml), and severe (2150 ± 2778ng/ml) disease. While statistical significance can be identified between the mild versus severe disease for D-dimer levels (*P* = 0.023; Fig. 1F), the spread in the values of D-dimer can make interpretation of individual cases difficult for necessary decisive action at clinics.

**Figure 2:**
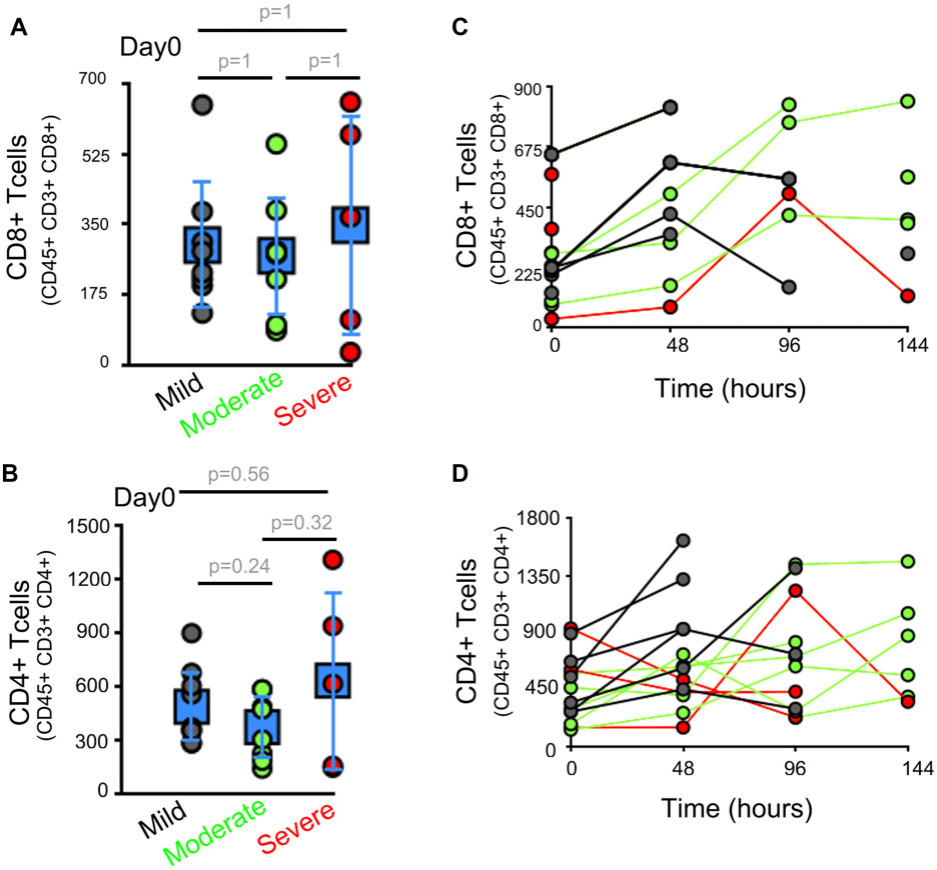
T-cell numbers are not different in COVID-19 patients with varying disease severity. (**A and B**) Dot plot represents FACS-based analysis of T-cell subtypes in peripheral blood, on Day 0, in patients with varying severity. CD8^+^ T cells (A; CD45^+^ CD3^+^ CD8^+^) and CD4^+^ T cells (B; CD45^+^ CD3^+^ CD4^+^). (**C and D**) Time-course analysis of CD8^+^ and CD4^+^ T cells in peripheral blood in patients with varying severity, at 48-h interval. CD8^+^ T cells (C; CD45^+^ CD3^+^ CD8^+^), and CD4^+^ T cells (D; CD45^+^ CD3^+^ CD4^+^). Error bars in A and B represents, average ± SD, Day 0 is the hospital admission day.

**Figure 3:**
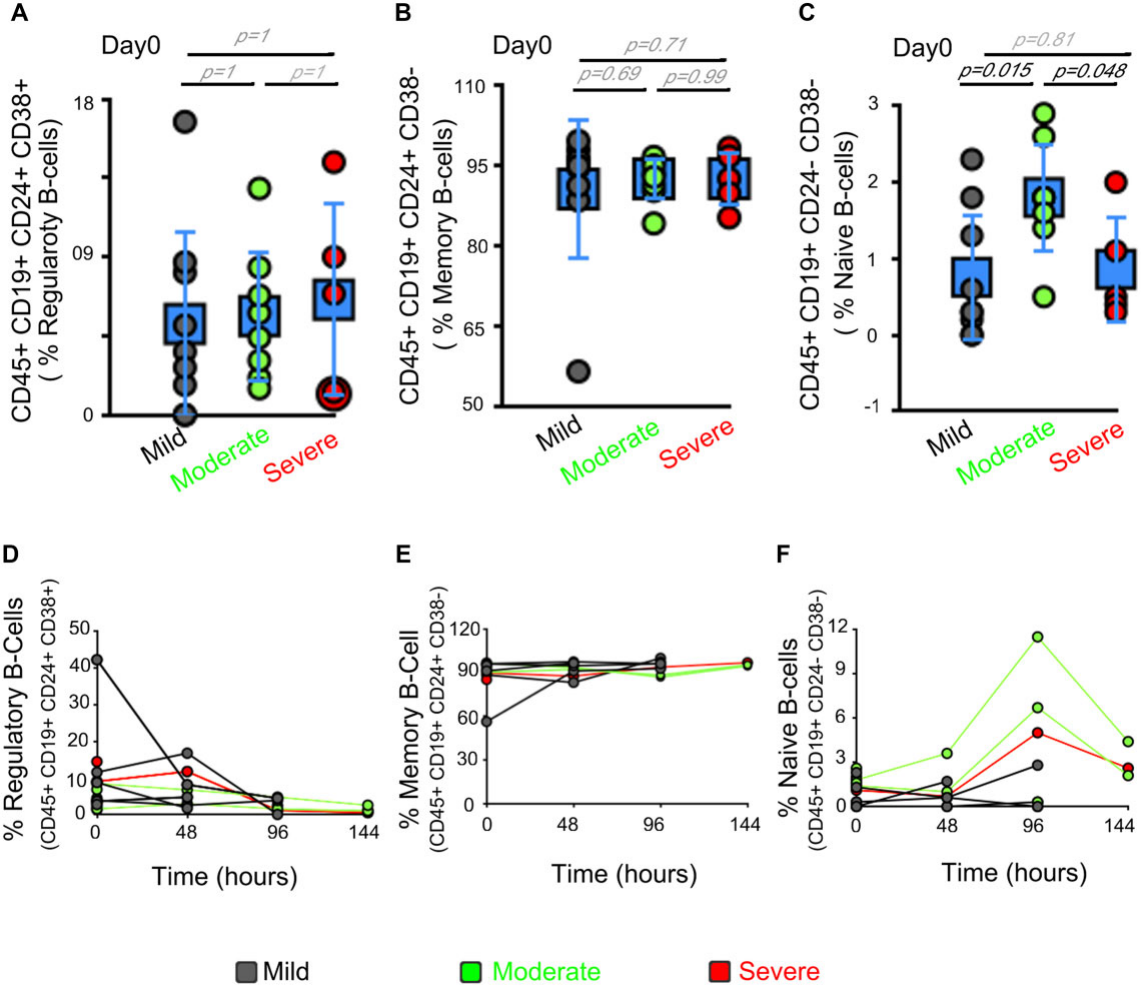
B-cell numbers are not different in progressive COVID-19 patients. (**A–C**) Dot plot represents percentage of B-cells subtypes, on Day 0, in patients with varying severity in peripheral blood. Regulatory B cells (A; CD45^+^ CD19^+^ CD24^+^ CD38^+^), memory B cells (B; CD45^+^ CD19^+^ CD24^+^ CD38^−^), and naive B cells (C; CD45^+^ CD19^+^ CD24^−^ CD38^−^). (**D–F**) Line graph represents changes in percentage of B-cells subtypes in peripheral blood of patients in patients with varying severity, at 48-h interval. Regulatory B cells (D; CD45^+^ CD19^+^ CD24^+^ CD38^+^), memory B cells (E; CD45^+^ CD19^+^ CD24^+^ CD38^−^), and naive B cells (F; CD45^+^ CD19^+^ CD24^−^ CD38^−^). Error bars (in square; A–C) represents average ± SD. Day 0 is the hospital admission day.

Earlier studies have highlighted that lymphopenia is a consistent phenotype in most COVID-19 patients [11, 12]. Lymphocyte levels (mostly T cells) were significantly lower in patients with severe versus mild disease and are thought to be useful as an early marker of disease severity [7, 13]. We hence reviewed the levels of T- and B lymphocytes in our study. We assessed the T-cell subsets of CD8^+^ T cells (CD45^+^ CD3^+^ CD8^+^) and CD4^+^ T cells (CD45^+^ CD3^+^ CD4^+^) by evaluating a standard combination of cell surface markers via Fluorescence activated cell sorter (FACS) based analysis. There was no significant difference in CD4^+^ or CD8^+^ T cells between the various severity groups on the day of hospital admission (Fig. 2A and B; *P* >0.05). Even in time-course analysis, at 48 h across their hospitalization period, there is no significant difference in the T-cell subsets between patients with severe, moderate, and mild disease progression (Fig. 2C and D). We analyzed the cell surface markers of the B-cell population via FACS-based analysis. B cells were divided into regulatory B cells (CD45^+^ CD19^+^ CD24^+^ CD38^+^), memory B cells (CD45^+^ CD19^+^ CD24^+^ CD38−), and naïve B cells (CD45^+^ CD19^+^ CD24^−^ CD38^−^). B-cell subtypes also did not show any significant difference between the various severity groups on Day 0, except for naive B cells in moderate group at Day 0 (Fig. 3A and C) or in the time-course analysis (Fig. 3D–F). We hence conclude that T cells or B cells are not substantially different in patients with mild or progressive COVID-19 disease.

### Severe patients show upregulation in most IL levels tested

**Figure 4:**
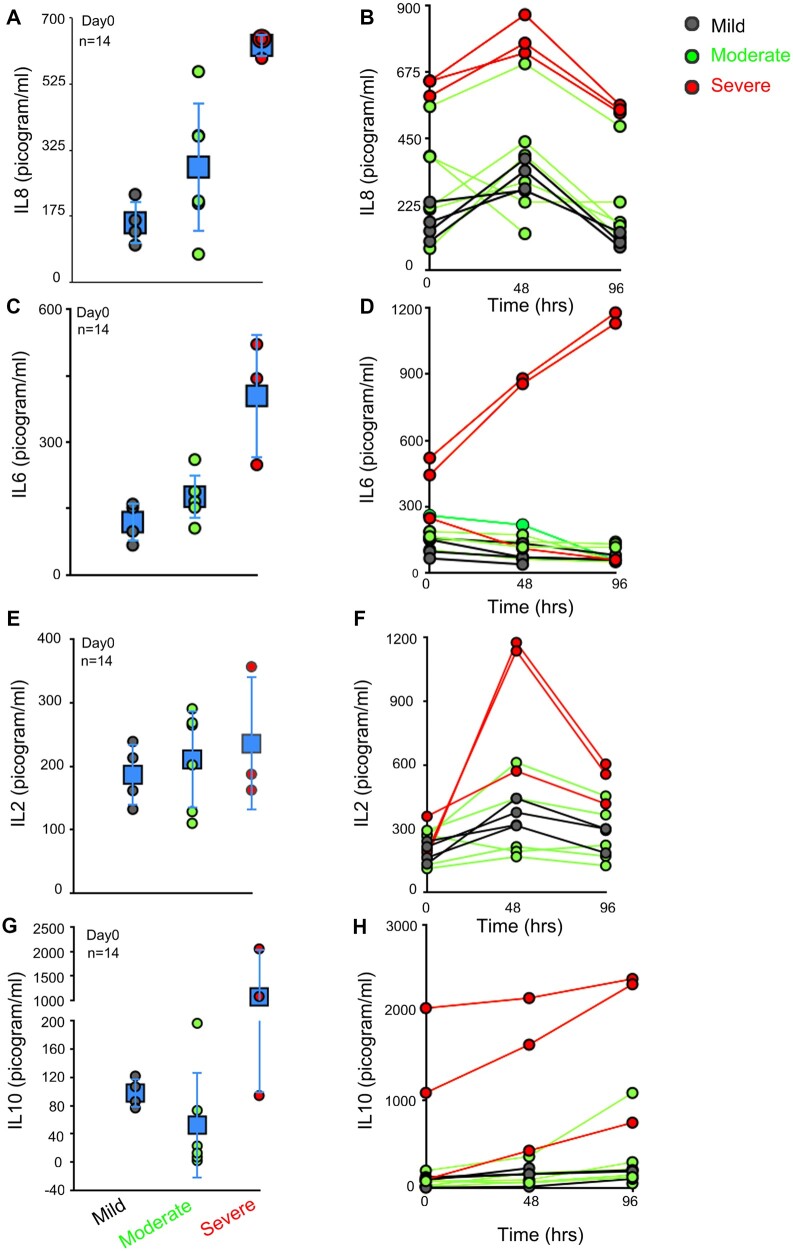
IL2, IL6, IL10, and IL8 are elevated in severe COVID-19 patients. (**A, C, E, and G**) Dot plot represents ELISA-based serum analysis on Day 0, in patients with varying disease severity, IL8 (A), IL6 (C), IL2 (E), and IL10 (G). Blue data points (in square) represent average ± SD. (**B, D, F, and H**) Line graph represents changes in serum values of IL8 (B), IL6 (D), IL2 (F), and IL10 (H) at 48-h interval. Error bars present average ± SD in A, C, E, & G. Day 0 is the hospital admission day.

We evaluated the cytokines in the serum of these patients to understand their temporal profiles. IL-8 levels were remarkably different even on Day 0 and during the hospitalization phase in patients with severe (626.53 ± 29.70 pg/ml) versus mild disease (203.48 ± 113.53 pg/ml) (Fig. 4A and B), unlike moderate disease (331.47 ± 210.34 pg/ml; Fig. 4A and B).

IL6 levels were also high in two out of three patients with severe disease tested in our pilot study (404.75 ± 140.31 pg/ml; Fig. 4C) compared to patients with mild disease (119.40 ± 44.32 pg/ml; Fig. 4C). The IL6 levels show further elevation during the hospitalization phase in two patients with severe disease (1153.83 ± 33.91 pg/ml at 96 h; Fig. 4D). While patients with severe disease but low IL6 level at hospitalization show further decrease in IL6 levels with time (compare red line graphs, Fig. 4D), suggesting an IL6-independent disease progression.

At the time of admission, IL2 levels were not significantly different between the various groups of patients (Fig. 4E; mild = 186.32 ± 48.38 pg/ml, moderate = 210.54 ± 76.84 pg/ml, or severe = 235.31 ± 105.67 pg/ml). IL2 increases significantly, but only transiently in two of the three patients with severe disease (Fig. 4F; 1156.35 ± 28.09 pg/ml) at 48-h post-admission compared to patients with mild disease (Fig. 4F; 361.088 ± 60.55 pg/ml). IL10 levels were also significantly elevated in two out of three patients with severe disease (1564 ± 689.35 pg/ml) versus mild disease (97.74 ± 20.41 pg/ml; compare Fig. 4G and H), while IL4 levels remain similar on Day 0 and in the time-course analysis for all 14 patients tested (Supplementary Fig. S2; 200–350pg/ml). TNF-α levels were below the detection level in all the patients tested.

### Lung biomarker, SP-D, can act as an early marker of disease severity

**Figure 5:**
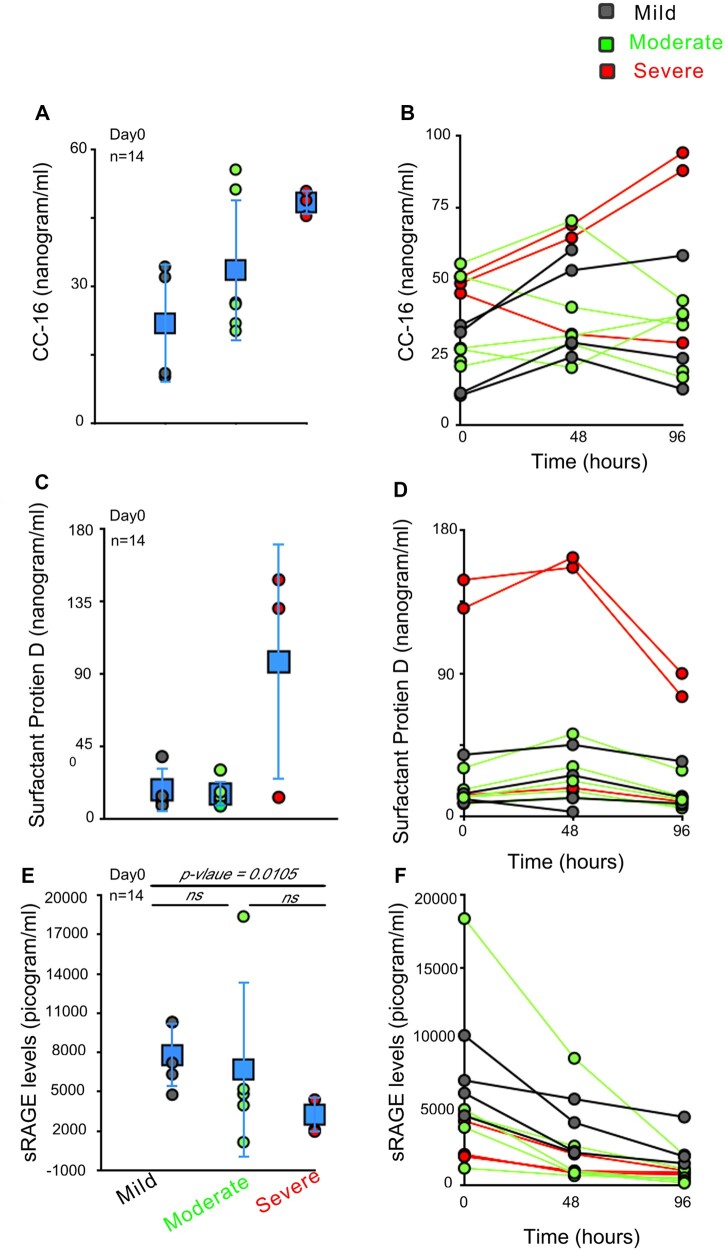
SP-D is upregulated in severe COVID-19 patients, unlike sRAGE. (**A, C, and E**) Dot plot represents ELISA-based analysis of serum levels of CC-16 (A); SP-D (C); and sRAGE (E) on Day 0 in patients with varying severity. Blue data points (in square) represent, average ± SD. (**B, D, and F**) Line graph represents changes in serum levels of CC-16 (B), SP-D (D), and sRAGE (F) levels in patients at 48-h interval. Error bars present average ± SD in A, C & E. Day 0 is the hospital admission day.

As COVID-19 is known to involve lung damage, we decided to evaluate the temporal profile of lung biomarkers in these patients. We used three lung markers for this study, which include Clara-Cell protein-16 (CC-16), also known as Uteroglobin; SP-D; and secretory receptor for advanced glycation end product (sRAGE). CC-16 is higher in patients with severe disease (48.41 ± 2.77 ng/ml, red data point, Fig. 5A) compared to the mild disease (21.89 ± 13.08 ng/ml, gray data points, Fig. 5A) but only on Day 0 (compare Fig. 5A and B). Unlike CC-16, SP-D levels are significantly elevated in two out of three patients with severe disease on Day 0 (139.79 ± 12.59 ng/ml) versus mild (19 ± 13.3 ng/ml) or moderate disease (15.72 ± 7.83 ng/ml) (Fig. 5C). SP-D levels remain high in two out of three patients with severe disease during the hospitalization period (Fig. 5D). sRAGE, on the other hand, shows a diametric profile. Serum levels of sRAGE are higher in mild disease with normal lungs (Fig. 5E, 7789.23 ± 2462.78 pg/ml) on the day of hospitalization compared to severe disease (3255.11 ± 1371.09 pg/ml) (Fig. 5E). sRAGE levels decrease with time (Fig. 5F), suggesting the differential ability of the infection-induced upregulation of sRAGE in most patients, also known for its anti-inflammatory role [14].

**Figure 6:**
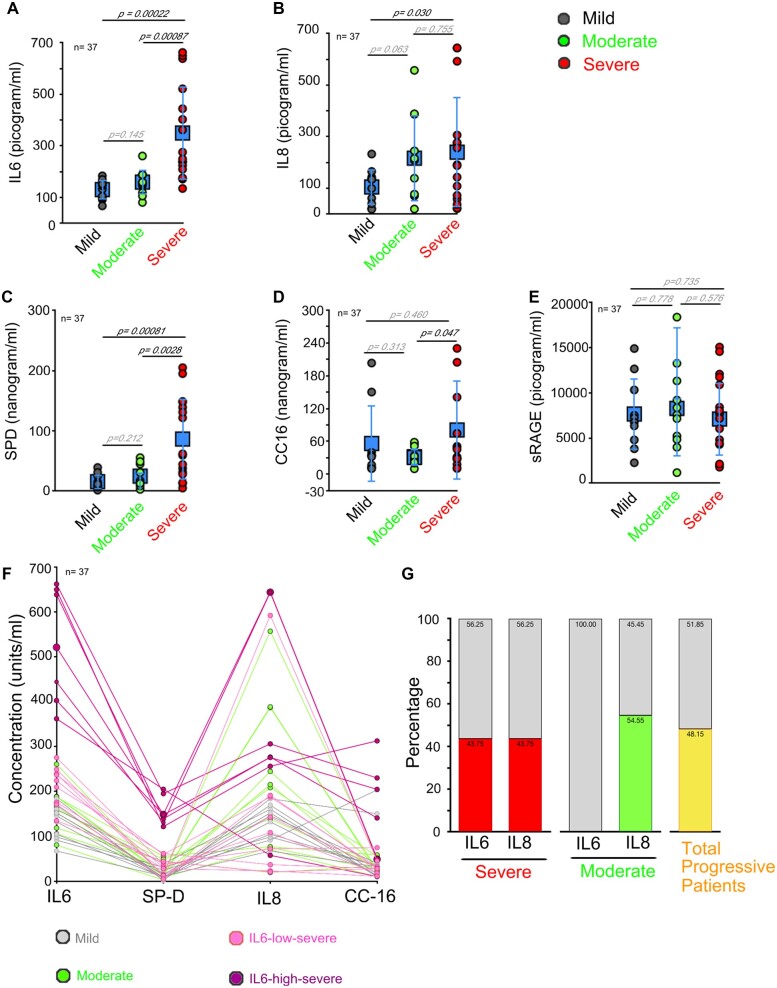
IL6 and IL8 can be used as early markers of progressive COVID-19 disease. (**A–E**) Dot plot represents ELISA-based analysis of serum levels of IL6 (A); IL8 (B); SP-D (C); CC-16 (D); and sRAGE (E) on Day 0 in COVID-19 patients. Blue data points (in square) in A–E represent average ± SD. (**F**) Line graph represents changes in serum levels of IL6, IL8, SP-D, and CC-16 per patient. Light-pink lines represent patients with IL6-low (300 pg/ml), dark-pink lines represent patients with IL6-high (>300 pg/ml), gray lines represent mild, and green lines represent moderate COVID-19 patients. (**G**) Bar graph representing percentage of COVID-19 patients identified with IL6-high (>300 pg/ml) or IL8-high (>200 pg/ml) in severe patients (red bars) or moderate (green bars) category, yellow bar represents percentage of total patients that fall in the progressive category with IL-high (>300 pg/ml) or IL8-high (>200 pg/ml) levels. Gray bars represents patients in each category with IL6-low (<300pg/ml) or IL8-low (<200 pg/ml) levels. Error bars present average ± SD (A-E). Day 0 is the hospital admission day.

Based on the finding from our temporal pilot study, we identified five serum markers namely IL6, IL8, SP-D, CC-16, and sRAGE, for analysis in a larger number of patients to evaluate their reliability as early markers of progressive disease. These markers were evaluated in a total of 37 patients, now only on Day 0. We find that both IL6 and IL8 were significantly upregulated in patients with severe disease compared to mild disease (Fig. 6A and B). IL6 levels are not different between mild (130.47 ± 38.22 pg/ml) and moderate disease (159.38 ± 48.73 pg/ml), but vary significantly in mild (130.47 ± 38.22 pg/ml) versus severe disease (349.69 ± 181.98, *P* = 0.00022; Fig. 6A) and between moderate and severe disease (Fig. 6A;*P* = 0.00087).

IL8 levels show significant difference only between mild (104.92 ± 69.61 pg/ml) and severe disease (239.57 ± 214.05 pg/ml; Fig. 6B;*P* = 0.030). We observed that patients with severe (239.57 ± 214.05 pg/ml; Fig. 6B) and moderate disease (216.35 ± 167.01 pg/ml; Fig. 6B) show a higher spread in serum IL8 levels. Higher variability between IL6 and IL8 might suggest a necessary molecular stratification of the progressive patients into “cytokine-dependent” and “cytokine-independent” categories.

Further of the three lung markers evaluated, serum SP-D levels are significantly upregulated in patients with severe disease (86.25 ± 67.74 ng/ml) compared to mild disease (15.34 ± 12.19 ng/ml; Fig. 6C;*P* = 0.00081). SP-D levels for moderate disease (24.57 ± 18.86 ng/ml) are not significantly different from mild unlike severe disease (Fig. 6C;*P* = 0.0028).

Again, CC-16 serum levels show significant spread, rather than differences between mild (56.16 ± 70.28 ng/ml), moderate (30.44 ± 15.59 ng/ml), and severe disease (80.79 ± 91.36 ng/ml) (Fig. 6D), while sRAGE failed to show any significant difference between mild, moderate, and severe disease conditions (Fig. 6E). Given the large dynamic range of the markers tested between the clinically defined mild, moderate, and severe categories of patients, we decided to evaluate their relative levels and trends per patient (Fig. 6F and Supplementary Table S2). For this, we divided the severe patients into IL6-low (<300 pg/ml, light pink line graphs, Fig. 6F and Supplementary Table S2) and IL6-high (>300 pg/ml, dark pink line graphs, Fig. 6F and Supplementary Table S2). This analysis reveals that all IL-6-high patients show upregulation in SP-D, IL8, and CC-16 levels. The IL6-low (<300 pg/ml) but clinically severe patients have IL8, SP-D, or CC-16 levels similar to the mild disease. Interestingly, clinically moderate disease shows upregulation in IL8 levels (>200pg/ml) but not in SP-D, CC-16, or IL6 levels (green line graphs, Fig. 6F). We hence propose that IL6 upregulation is associated with higher inflammatory status and damage in COVID-19 patients. Only IL8-high (>200 pg/ml) levels might serve as marker of cytokine-induced moderate disease. Our data also indicate that IL8 upregulation alone can lead to progressive disease, but the extent of inflammation-induced damage is less in these patients. Using >300 pg/ml as the cut-off for IL6 and >200 pg/ml IL8 in our study, 50% of the patients with progressive (moderate or severe) COVID-19 can be identified for early therapeutic intervention (Fig. 6G and Supplementary Table S2). Importantly, our data also pinpoint that 50% of the patients in our study had rather cytokine-independent progression. We propose that only patients with IL6-high (>300 pg/ml) should be considered for early intervention with the available IL6-inhibitor drugs like Tocilizumab. Also, not only Tocilizumab but even immunomodulators should be avoided in patients with IL6-low (<300 pg/ml) and IL8-low levels (<200 pg/ml) as they might be progressive due to the direct influence of the virus, independent of host-immune responses.

## Discussion

COVID-19 infection has challenged the healthcare system globally for more than 2 years now. The initial viral genome sequencing efforts have identified that there are several mutations in the receptor-binding domain of the Spike (S)-proteins of the SARS-CoV-2. These mutations make this COVID-19-causing virus highly infective by increasing affinity for the Angiotensin-Converting Enzyme (ACE)-2 receptors [15]. SARS-CoV and the SARS-CoV-2 viruses use the same S-protein to infect the host cells [15–17] yet, they do not show significant antibody cross-reactivity. This has been attributed to the mutations in the antigenic epitopes of the S-protein with 92.7% unique epitopes contributed by the nonconserved region. SARS-CoV-2 has 20 unique epitopes as compared to SARS-CoV in the S-protein region, and 40 epitopes from SARS-CoV are not present in SARS-COV-2, only 5 epitopes are shared [1, 18]. Due to this, SARS-CoV-specific antibodies do not bind to the S-protein of SARS-CoV-2, and there is a need to develop SARS-CoV-2-related antibodies and vaccines independently. Currently, it is expected that available COVID-19 vaccines may not prevent the infections altogether but can mitigate or block lung involvement in most patients [19]. Vaccine manufacturing to cater to the entire population has been challenging for most countries and is turning out to be the current rate-limiting step in allowing us to resume the requisite socio-economic normalcy. One of the challenges across the globe has been the flooding of hospitals with COVID-19 patients and the shortage of critical care beds for the sick. Hence identifying additional avenues to intervene post-infection will be needed to tackle this pandemic. Knowledge of who is likely to progress to severe disease will help triage patients better. Similarly, identifying patients early in the course of the disease who will benefit from immunomodulators or newer therapies will help with early intervention and better outcomes. So far, this has been done using clinical characteristics which may be too late like the degree of hypoxia or not very discriminatory factors like age or presence of comorbidities.

Many COVID-19-related studies done in early 2020 have improved the understanding of the SARS-CoV-2 virus and have facilitated the rapid development of therapeutic interventions. However, several molecular aspects about COVID-19 infection and disease severity remain poorly resolved. Severe COVID-19 is characterized by hyper-inflammation leading to “cytokine storm” which results in widespread inflammatory damage, particularly to the lung. We hence studied select cytokines and lung injury markers to evaluate if they can anticipate COVID-19 patients progressing to severe disease. We evaluated serum levels of select cytokines and lung markers at 48-h intervals from the time of admission through the duration of hospitalization at our tertiary care center. Our study has identified early biomarkers that can pinpoint COVID-19 patients likely to progress to severe disease triggered by host-immune responses. Such patients could be considered for timely therapeutic intervention with available immunomodulator drugs or precision medicines.

ILs can act as autocrine and paracrine pro-inflammatory or anti-inflammatory molecules. ILs and most other cytokine levels in our body are highly dynamic and increase in several pathological conditions [20]. Several cells are involved in cytokine secretions, these include fibroblasts, keratinocytes, vascular endothelial cells, pericytes, mast cells, macrophages, dendritic cells, and T and B cells. Cytokine storm is a term coined to describe the secretion of high levels of several cytokines that can increase inflammation [21]. SARS-CoV-2 virus-induced host-immune responses can induce lung damage, thrombosis, multi-organ failure, and death in COVID-19 patients [21–23]. Currently, clinics are using immunomodulators or precision drugs only if severe symptoms appear. Our data suggest that progressive patients can be identified even in the early phase of the disease for timely intervention with available immunomodulators. In our study, ∼50% of the severe patients show upregulation of most of the ILs tested (except IL4), much before the manifestations of severe clinical phenotypes. We find that patients with high IL6- levels (>300 pg/ml) at hospitalization are distinct and may be wired for a severe manifestation of the disease.

IL6 has been identified as a progressive disease marker by several other studies [24–27]. A study from Slovakia suggests that IL6 levels of >24 pg/ml are a reliable indicator of progressive disease [24], whereas studies from China suggest that IL6 levels of >32–37 pg/ml represent progressive disease [26, 27]. We find that Indian patients with mild disease (no hypoxia or lung involvement) show much higher IL6 levels, that is 130.47 ± 38.22 pg/ml, while severe patients (<90% SpO_2_) demonstrate average IL6 levels of 349.69 ± 181.98 pg/ml (Fig. 6A, *P* = 0.00022). Hence, the proposed IL6 cut-off for severe disease prediction is thus much higher in India and might represent geographical variations in immune status. This may mean each country needs to define its own mild versus severe cut-off of the biomarkers.

Progressive COVID-19 condition caused mainly by cytokine storm, if identified early, and treated in time, can prevent damage to vital organs and improve outcomes. There are several inhibitor drugs available to treat cytokine storm, including ACE inhibitors, angiotensin II receptor blockers, and corticosteroids. The inhibitor of IL6, Tocilizumab, has also been explored as a therapeutic agent to overcome cytokine storm in severe COVID-19 patients with varying outcomes [28, 29]. We suggest that severe patients should be further stratified based on their IL6 and IL8 levels, into the “cytokine-dependent or “cytokine-independent” categories (Fig. 6F and G; Supplementary Fig. S2). Immunomodulators or precision drugs must be restricted to the “cytokine-dependent” category of patients thus obviating potential side effects of these drugs.

In our modest but random sampling of COVID-19 patients in this study, ∼50% of the severe category of patients show cytokine-independent progression (Fig. 6F–G and Supplementary Table S2). Severe patients with low levels of IL6 (<300 pg/ml) do not show upregulation in IL8, CC-16, or SP-D levels in our study. We hence propose that these patients are progressive without the inflammatory host-immune response or cytokine storm and are not suitable for immunomodulators or precision medicine-based intervention. Also, moderate patients often do not show upregulation in IL6 levels but can have upregulated IL8 levels only (Fig. 6F–G). Such patients should not be given IL6-inhibitor drugs like Tocilizumab.

The selective upregulation of IL8 in moderate patients suggests that secretion of IL6 and IL8 are independently regulated. Molecules like Trek-1 (a potassium channel) [30] and the naturally occurring compound called humulene [31] can exclusively regulate the secretion of IL6 or IL8, respectively. The differential molecular regulation of IL6 and IL8 might be responsible for severe/moderate versus mild COVID-19 progression. Critical molecular regulators of IL6 and IL8, if identified, can provide a better hold on this disease. Several open questions need to be resolved for this. Are the levels of Trek-1 differential between severe, moderate, and mild patients? IL6 expression is regulated by Toll-Like Receptors (TLRs) and other Pathogen Recognition Receptor (PPR) families mainly via Nuclear Factor kappa B (NF-kB) signaling [32], nuclear factor of activated T cell [33], or activator protein-1 [34]. Are the regulators of these signaling arms differentially expressed in progressive patients? or are the suppressor of cytokine signaling [35] differential? Is IL6 upregulation more consequential than IL8 upregulation? If yes, Why? Also, we notice that all patients with severe disease manifestation and even some moderate patients show >50% lung damage, despite these lung-specific biomarkers (SP-D or CC-16) are not elevated in the serum of IL6-low category of patients (Fig. 6F compare dark-pink line graphs with light-pink and green line graphs; Supplementary Table S2). Does this represent specific type of lung damage via IL6 and/or other associated cytokines or is it due to some pre-existing lung condition? We have also noticed that few patients do not show any lung damage, using X-ray, but have moderate disease with high IL8 levels (>200 pg/ml) (Supplementary Table S1). What leads to oxygen deprivation in such patients? These preliminary observations need closer investigation(s). Initially, it was thought that the severe manifestation of this disease is due to age-mediated changes in the molecular profiles or due to co-morbidities. However, in the second wave of COVID-19 infection, these correlations did not hold, suggesting a constantly evolving picture. A lot still needs to be done at the molecular level to understand the molecular changes responsible for severe disease progression in patients, independent of their biological age.

Constantly mutating genome of SARS-CoV-2, the increasing infectivity, and their severity make it challenging to act and think ahead of this virus. It is increasingly evident that many COVID-19-infected patients enter the progressive phase of the disease due to virus-induced host-immune reactions. Our study suggests that IL6 and IL8 levels must be considered for molecular stratification of COVID-19 patients, irrespective of their clinical stratification to pre-empt “cytokine storm”-dependent disease progression. Currently, most countries manage severe conditions only on the onset of the symptoms, making it challenging to treat severe patients effectively to improve outcomes. IL6 and IL8 levels, rather than CRP levels, should be monitored to identify severe or moderate patients from mild for necessary and timely intervention with available immunomodulators or precision drugs. Importantly, patients with IL6-low (<300 pg/ml) and IL8-low (<200 pg/ml) might be progressive due to direct virus-induced damage and should not be subjected to further immune suppression via immunomodulatory drugs or precision medicines. It might be useful to evaluate if this subset of patients could rather benefit from antiviral drugs. Currently, based on the initial findings [36], all patients with hypoxia (moderate or severe disease) are administered Dexamethasone (Supplementary Tables S1 and S2). Our findings suggest that this current clinical practice needs a closer and critical evaluation. As we learn to live with this virus and its ever-changing infectivity and severity pattern, resolving our clinical approach further will be useful in improving outcomes. However, our proposed IL6-high and IL8-high-based patient stratification need a larger study for validation and determination of a more precise cut-off for the cytokines-based stratification. We envisage that our proposed stratification based on cytokine signatures may not be limited to SARS-CoV-2 infections only. It might cover a larger canvas of viral or bacterial infectious agents known for causing cytokine storm-mediated organ damage and deaths. Nonetheless, this proposition needs further experimental validation.

## Methods and materials

### Samples

Venous blood was collected from Polymerase Chain Reaction (PCR)-positive COVID-19 patients in Ethylenediamine tetraacetic acid (EDTA)-vaccutainers and clotting tubes (1 ml each). All blood samples were immediately stained for cell surface markers for FACS-based analysis. Sample aliquoting as well as staining was done in a BSL2 cabinet with Personal Protective Equipments (PPEs). Serum samples were aliquoted and stored at −80°C freezer for subsequent enzyme-linked immunosorbent assay (ELISA)-based analysis. COVID-19 safety guidelines and protocols were followed for all wet lab-based work using patient samples.

### Cell staining for flow cytometric analysis

The antibody capture beads, unstained beads (negative beads and positive beads), and single-stained beads (negative beads + positive beads) were used for color compensation as per the manufacturer’s protocol (552843 BD^TM^ CompBead, Anti-Mouse). Antibody mix was prepared using five different antibodies according to their respective recommended concentrations. Precisely, 50 μl blood was added to it in 5 ml FACS™ tubes with antibodies. The tubes were incubated for 15 min in the dark at room temperature. To lyse RBC, 450 μl of 1X BD FACS™ lysing solution was added and again incubated for 15 min at room temperature. Antibody mix includes CD45 (563204, Mouse, BD Biosciences), CD38 (555462, Mouse, BD Biosciences), CD24 (Mouse, 555427, BD Biosciences), CD66B (Mouse, 561650, BD Biosciences), CD19 (Mouse, 560728, BD Biosciences). No-spin lyse, no-wash protocol was followed to avoid aerosol generation during sample processing. Samples were processed in a BSL2 cabinet with appropriate PPEs.

### Enumeration of immunocytes using BD TruCount™ tubes by flow cytometry

Cell Staining: Ten microliter of individual antibodies were added into BD TruCount™ tubes (Catalog No. 340334) containing the beads according to their respective recommendations to product insert. Precisely, 50 μl blood was added to it. It was incubated with a recommended concentration of different antibodies for 15 min in dark at room temperature. To lyse RBC, 450 μl of 1X BD FACS™ lysing solution was added and again incubated for 15 min at room temperature. Antibodies mix includes CD45, CD3, CD4, and CD8 (Mouse, 340499, BD Biosciences). Samples were processed in a BSL2 cabinet with appropriate PPEs. The scatter and fluorescence data of unstained and stained cells were acquired on a BD FACS Fortessa X-20™ flow cytometer using FACS™ diva software version V.8.0.3 (San Jose, USA).

The gating strategy applied to quantify the percentage of CD45, CD38, CD24, CD66B, and CD19 cells is given in Supplementay Fig. S3. The absolute counts were obtained according to the BD TrueCount™ Tube product insert, and gating strategy applied to enumerate the absolute number of each cell type is given in Supplementay Fig. S4. For example, to enumerate the absolute count of the cell population (A), by dividing the number of positive cell events (X) by the number of bead events (Y), and then multiplying by the BD TruCount™ bead concentration (N/V, where *N* = number of beads per test* and *V* = test volume). A = X/Y × N/V.

### ELISA

Serum samples were diluted 1:5 times for ELISA analysis. Standards were prepared according to the manufacturer’s protocol. ELISA kits were used to prepare the samples according to each antibody. Cytokines analyzed include IL2 (0017212, BD Biosciences), IL6 (550799, BD Biosciences), IL8 (550999, BD Biosciences), IL4 (550614, BD Biosciences), TNF-α (550610, BD Biosciences), and IL10 (550613, BD Biosciences) and Lung markers include Uteroglobin (DUGB00, R&D Systems), SP-D (DSFPD0, R&D Systems), and sRAGE (DRG00, R&D Systems). Samples were processed in a BSL2 cabinet with appropriate PPEs.

### Regulatory approvals

The study was approved by the Institutional Ethics Committee at St. John’s Medical College and Hospital (IEC ref. No. 144/2020, dated 22 May 2020). This project was reviewed by Review Committee on Genetic Manipulation (RCGM) for Biosafety approval, Department of Biotechnology (DBT), Goverment of India (GoI) (on 27 July 2020) and approved for the required wet-lab work with necessary safety compliances (Ref no. BT/17/006/96-PID, dated 2 July 2020).

### Informed consent

Venous blood and serum samples of patients were collected by the clinical team on the day of admission and at 48 h after taking written and informed consent.

## Supplementary Material

bpac028_Supplementary_Data

## Data Availability

All the experimental data underlying this article are available in the article and in its online supplementary material. Patients' clinical data underlying this article cannot be shared publicly as per the ethics and privacy requirement of individuals that participated in the study.
