# Supplementary material for: Molecular markers for early stratification of disease severity and progression in COVID-19
Source: Biol Methods Protoc. 2022 Nov 2;7(1):bpac028. doi: 10.1093/biomethods/bpac028 (PMC9731223; doi:10.1093/biomethods/bpac028)
Supplement: bpac028_Supplementary_Data [file bpac028_supplementary_data.zip › Supplementary Table 1_Dec2021.pdf]

Supplementary Table 1: COVID-19 Patients clinical details

	Serial no.	Age & Gender	Gender	Symptoms on Day0	Treatment<br>Dexamethasone	Remedesivir	Tocilizumab	Chest X-ray (CXR)	Symptom to Hospitalization	At SJMH	COVID category	O2 levels?	Remark
	Patient1	25	Female	Co-morbidities: none<br><br>Symptoms:fever, cough and breathlessness since 10 days	Yes	No	No	b/l infiltrates <50%	10	3	mild covid, stable, uneventful course	>94%	
	Patient 2	35	Male	Co-morbidities: HYPERTENSION, DIABETES MELLITUS<br><br>Symptoms: COUGH BREATHLESSNESS SINCE 3 DAYS	No	No	No	Normal	3	3	MILD COVID	>94%	
	Patient 03	22	Female	Co-morbidities: PCOS<br><br>Symptoms:fever cough since 9 days , anosmia, dysguesia since 4 days	No	No	No	NORMAL	10	3	Mild covid	>94%	
	Patient 4	48	Male	Co-morbidities: DEPRESSION, SAH POST SURGERY, HYPERTENSION Symptoms: 2days LOSS OF APPETITE ,DRY MOUTH AND FATIGUE. NO FEVER, COUGH OR BREATHLESSNESS	No	No	No	NORMAL	3	7	MILD COVID	>94%	
	Patient 5	??	Female	Co-morbidities:NONE<br><br>Symptoms:TIREDNESS ANOSMIA AND DYSGUESIA SINCE 3 DAYS	No	No	No	NORMAL	3	7	UNEVENTFUL, MILD COVID	>94%	
	Patient 06	46	Male	Co-morbidities: NONE<br>Symptoms: Dry cough x 1 week, Easy fatiguability x 3 days	No	No	No	NORMAL	7	10	MILD COVID	>94%	
	Patient-19	52	Male	MILD COVID, UNEVENTFUL	No	No	No	NORMAL	5	15	MILD COVID	>94%	
	Patient 21	68	Male	Co-morbidities: NONE<br>Symptoms: FEVER AND COUGH SINCE 4 DAYS	No	No	No	60% infiltrates	4	12	MILD COVID, UNEVENTFUL	>94%	
**Mild COVID**	CVD-32	68	Female?	COVID-19: Mild COVID<br>Co-morbidities: DM , HTN, IHD<br>Others: ? LV dysfunction- recent ACS-STEMI presently NSTEMI, Burning pain in chest and upper abdomen x 20 days<br>b. Constipation x 4 days<br>c. Vomiting x 2 days	No	No	No	NORMAL	20	6	MILD COVID	>94%	
	Patient 09	74	Male	Co-morbidities: HYPERTENSION, T2DM, IHD<br>Symptoms: c/o generalized fatigue x 10 days, Myalgia x 10 days	No	No	No	B/L MID AND LOWER ZONE OPACITIES WHICH WORSENED TRANSIENTLY FROM 30% TO 50% INVOLVEMENT AND IMPROVED AT DISCHARGE	10	12	MILD COVID		
	CVD-41	30	Female	DCLD-CHILD PUGH A<br>OGD-PENDING<br>SEIZURES-SECONDARY TO ? HYPONATREMIA<br>CT BRAIN-REFUSED<br>HYPONATREMIA EVALUATION-PENDING,<br>MALENA SINCE 21/06/2021 , HEMATURIA 1 EPISODE ON 21/06/2021				22/6/21 : CXR : B/L infilterates 30% involvement	1	4	MILD COVID	>94%	**Not included**
	CVD-43	36	Female	QUADRIPARESIS UNDER EVALUATION-PROBABLE GB SYNDROME<br>2. HRV DISEASE - TREATMENT FAILURE<br>3. PANCYTOPENIA<br>4. MILD COVID<br>5. ORAL CANDIDIASIS,Cough with expectoration since 2 months Dyspnea on excertion and inability to eat with odynophagia since 15 days	-	-	-	25/6/21 : Left diffuse infilterates 30-40% involvement	15	5	MILD COVID	>94%	**Not included**

Moderate COVID	PAtient 7	53	Female	Co-morbidities: k/c/o T2DM since 5 years,HTN x 3 years ,Hypothyroidism<br><br>Symptoms: Breathlessness on exertion x3 days<br>fever x 3 days<br>cough x 3 days<br>blood tinged sputum x 2 days	Yes	No	No	MILD LOWER ZONE INFILTRATES ~50% INVOLVEMENT	3	4	MODERATE COVID	90-93%	
	Patient 11	50	Male	Co-morbidities:<br>1.UNCONJUGATED HYPERBILIRUBINEMIA<br>2.MODERATE COVID<br>3.RBBB WITH EPISODIC BRADYCARDIA<br>4. ETHANOL ABUSE -? CLD<br>5.THROMBOCYTOPENIA<br><br>Symptoms: Tiredness and myalgia x 4 days<br>Dyspnoea x 2 days	Yes	Yes	No	Cxr: 60% lung involvement upper mid and loer zone opacities +	4	6	MODERATE COVID	90-93%	**Not included**
	Patient 12	81	Male	Co-morbidities: DM/HTN/IHD<br><br>Symptoms: The patient presented with a 4 day history of fever, cough and breathlessness.	Yes	No	No	NORMAL	5	8	MODERATE COVID	90-93%	
	Patient 13	74	Male	Co-morbidities: BRONCHIAL ASTHMA<br><br><br><br>Symptoms:BREATHLESSNESS SINCE 3 DAYS	Yes	No	No	CXR: LEFT LOWER ZONE INFILTRATES WITH CP ANGLE BLUNTIING 15-30 % INVOLVEMENT.	3	24	MODERATE COVID	90-93%	
	PAtient 17	57	Male	Co-morbidities: HYPERTENSION,<br>Symptoms: Cough x 4 days. Breathlessness x 4 days.	Yes	No	No	4/9/21 : CXR : Supine AP, poor expiratory film, apparent cardiomegaly, Bilateral infilterates with >50% involvement	10	17	IMPROVED, MODERATE COVID RECOVERED AND DISCHARGED, STAY UNEVENTFUL	90-93%	
	Patient 18	45	Male	Co-morbidities:<br>HTN<br>CKD<br>STEROID INDUCED HYPER GLYCEMIA<br>Symptoms: fever X 10 days	Yes	No	No	40-50% infiltrates	4	6	MODERATE COVID, IMRPOVED WITH STEROIDS AND OXYGENATION	90-93%	
	Patient 23	53	Male	Co-morbidities: NONE<br><br>Symptoms: fever x 3days<br>breathlessness x 1 day<br>fatigue and giddiness	Yes	No	No	MILD LOWER ZONE INFILTRATES, <50%	3	12	MODERATE COVID	90-93%	
	Patient 25	56	Male	Co-morbidities: TYPE 2 DIABETES MELLITUS<br><br>HYPERTENSION<br>Symptoms:<br>Fever since 5 days<br>Cough with breathing difficulty * 5 DAYS	Yes	Yes	No	BILTERAL MINIMAL LOWER ZONE INFILTRATES<br>10-20%	5	10	MODERATE COVID	90-93%	
	CVD-26	53	Male?	newly diagnosed DM. cough, fever, mayalgia for 3days	Yes	No	No	11/6/21 : CXR : B/L infilterates 40-50% involvement CT score - 12/25<br>Peripheral B/L ground glass opacities with consolidation.	3	4	MODERATE COVID	90-93%	
	CVD-35	31	Male?	GENERALISED LYMPHADENOPATHY	Yes	No	No	18/6/21 CXR : B/L infilterates 30-40 % involvement	20	5	MODERATE COVID	90-93%	
	CVD-38	49	Female	fever and myalgia since 1 month. C/O headache * 1 week. C/o breathlessness since 3 days. Patient tested positive 8 days, TYPE 2 DIABETES MELLITUS HYPERTENSION BRONCHIAL ASTHMA DIABETIC FOOT S/P AMPUTATION STEROID DEPENDENCE	Yes	No	No	18/6/21 : CXR : 30-40 % involvement	8	5	MODERATE COVID	93%	
	Patient 10	85	Male	Co-morbidities: HYPERTENSION<br><br><br><br>Symptoms: SHORTNESS OF BREATH, 3days. COUGH, 2days	Yes	No	No	NORMAL	3	8	MODERATE COVID	90-93%	

**Severe COVID**	Patient 8	72	Female	Co-morbidities: NONE.<br>Symptoms: Symptoms: Fever x 10 days, Cough x 10 days, Exertional dyspnoea x 2 days	Yes	No	No	CXR: B/L NON HOMOGENOUS OPACITIES 40-50% INVOLVEMENT	10	10	SEVERE COVID	<90%	
	COVID14	25	Male	Co-morbidities: NONE<br><br>Symptoms: Patient came with complaints of:<br>1. Fever x 2 days<br>2. Cough x 2 days<br>3. Breathlessness x 2 days	Yes	Yes	No	CXR: B/L UPPER MID AND LOWER ZONE OPACITIES 60% INVOLVEMENT	4	14	SEVERE COVID	<90%	
	Covid15	59	Male	T2DM x 1year, on OHA. Currently came with reduced appetite and breathing difficultyGeneralised weakness x 5months Breathing difficulty x 1week	Yes	No	No	3/9/2020 CT KUB (PLAIN)<br>IMP:Bilateral bulky kidneys with perinephric and periureteric fat stranding, bilateral hydroureteronephrosis (R >L) with urothelial thickening - s/o Pyelonephritis.	7	25	Severe Covid with TB	<90% (83%)	
	Patient 16	70	Female	Co-morbidities: k/c/o DM and HTN x 5 years. Hypothyrodism x 6 years.<br>Symptoms: Breathlessness x 1 week, Cough x 1 week , Decreased appetite x 1 week	Yes	Yes	No	CXR: 40% INVOLVEMENT MID AND LOWER ZONE INFILTRATES	7	17	Severe COVID	<90%	
	Patient 20	31	Male	Co-morbidities: COVID -19 PNEUMONIA WITH TYPE 2 RESPIRATORY FAILURE<br>B/L SUBCUTANEOUS EMPHYSEMA S/P B/L ICD<br>VAP / BSI - ACINETOBACTER IN REFRACTORY SEPTIC SHOCK<br>Moderate to severe depressioN<br><br>Symptoms: C/o fever, body pain and headache x 5 days prior to admission on 3/9/2020	Yes	No	No	EXTENSIVE GROUND GLASSING	5	Day6 shifted to ICU	HRCT: CORADS 6 80-90% LUNG INVOLVEMENT	<90%	
	Patient 22	61	Male	Co-morbidities: HTN<br>TYPE 2 DM<br>Symptoms: c/o fever x 1 week ,<br><br>breathlessness 3 days<br><br>cough x 3 days	Yes	Yes	No	EXTENSIVE SHADOWS 70-80% INVOLVEMENT	7	25	DIAGNOSIS: SEVERE COVID WITH POST COVID SEQUELAE	<90%	
	Patient 24	80	Male	Co-morbidities: ACUTE ON CHRONIC NON HEMORRHAGIC INFARCT - ACA TERRITORY<br>ACUTE KIDNEY INJURY S/P HD<br>DM * 15 YEARS<br>Hypertension x 15 years<br><br>Symptoms: Cough x 5 days<br>Breathlessnessx 5 days	Yes	Yes	No	HRCT: CORADS 6 ~70% LUNG INVOLVEMENT (16/25, CT)	5	7th day, Death	SEVERE COVID	<90%	**Not included**
	CVD-27	45	Male?	cough for 4 days and breathlessness for 1 day and also had loose stools and vomiting for 2 days	Yes	Yes	Yes	13/8/21 : cxr : b/l Mid zone and lower zone infilterates 30-40 %, CT PA; on 23/6/21 revealing diffuse ground glass opacitiess with crazy paving in bilateral lung fields,CT involvementcore of 24/25.	4	19	SEVERE COVID	<90%	**Not included**
	CVD-28	68	Female	PNEUMONIA WITH ARDS<br>RHEUMATOID ARTHRITIS ASSOCIATED WITH INTERSTITIAL LUNG DISEASE<br>yspnoea since 1 day<br>Fatigue since 1 day. Dry cough since 1 day	Yes	No	No	11/06/21 Chest Xray-B/L patchy infilterates 70% lung involvement	1	6the day , Death	SEVERE COVID	<90%	
	CVD-30	38	Female	TYPE 2 DIABETES MELLITUS<br>HYPOTHYROIDISM<br>BRONCHIAL ASTHMA<br>OBSTRUCTIVE SLEEP APNOEA,<br>Sore throat x 3-4 days<br>Breathlessness x 1 day<br>Wheeze +<br><br>Breathlessness x 2 days<br>no h/o fever, cough, loose stools<br>h/o irregular menses x 2 months	Yes	Yes	No	B/L costophrenic and left cardiophrenia angle blunting. Opacities on left lower and upper zone. Pleural effsuion minimal to moderate, left more than right. 60 - 70 % involvement	4	11	SEVERE COVID	<90%	

	CVD-33	39	Male	Diabetes Mellitus on OHA, not compliant,Cough x 2 days<br>Fatigue x 1 day	Yes	?	?	8/7/21 : CXR : B/L diffuse infilterates with >90 % involvement, 26/26 HRCT	2	28th Death	POST COVID ILLNESS. Death	<90%	
	CVD-34	45	Male	Steroid induced hyperglycemia,Cough since 4 days/ Dyspnea since 4 days	Yes	Yes	No	19/6/21 : CXR : B/L infilterates right > left 60-70 % involvement	4	8	SEVERE COVID	<90%	
	CVD-36	65	Female?	EVERE COVID (DISCHARGE AGAINST MEDICAL ADVICE)<br>URINARY TRACT INFECTION<br>TYPE 2 DIABETES<br>HYPERTENSION<br>ISCHEMIC HEART DISEASE WITH NORMAL EF<br>COPD AND OSA ON LTOT AND CPAP<br>HYPOTHYROIDISM<br>DYSLIPIDEMIA,Fever x 2 days<br>Breathlessnss x 2 days<br>Vomiting x 2days	Yes	No	No	17/6/21 : Right diffuse 25% infilterates 9/40	2	8	SEVERE COVID	<90%	
	CVD-37	57	Male	Cause of death: Refractory septic shock<br><br>COVID Pneumonia with ARDS<br><br>Upper and lower GI Bleed (Mallory weiss tear ; Caecal and rectal ulcers)<br>Diabetes mellitus<br><br>Hypertension<br><br>Coronary Artery Disease<br><br>B Thalassemia trait	Yes	Yes	Yes	9/7/21 : CXR : B/L infilterates 90-95% involvement	3	22nd Day death	SEVERE COVID	<90%	
	CVD-39	61	Female	T2DM<br>HbA1C- 12.2<br>3. HYPOTHYROIDISM<br>TSH - 10.7<br>4. ANAEMIA - IDA<br>5. DYSLIPIDEMIA<br>6. ORAL CANDIDIASIS<br>7.?LARYNGEAL CANDIDIASIS<br>8.CHRONIC SINUSITIS	Yes	Yes	No	17/7/21 : CXR : B/L infilterates 80%	6	33	SEVERE COVID	<90%	
	CVD-40	77	Female	PULMONORY THROMBOEMBOLISM (22/06/21)<br><br>RECENTLY DETECTED STEROID INDUCED HYPERGLYCEMIA (A1C = 6.3) ,Fever x 3 days<br>Dysuria x 3 days	Yes	Yes	No	18/7/21 : CXR : B/L infilterates with 60 % involvement<br>c. CT - HRCT-21/6/21- Partial/chronic pulmonary thrombo embolism involving the left lower lobar subsegmenta,l branches., 24/25, HRCT, >90% infiltrates<br>Features of typical COVID Pneumonia - CORADS 6 with CT involvement score of 24 /25 (severe).	6	18	SEVERE COVID	<90%	
	CVD-42	81	Female	TYPE 2 RESPIRATORY FAILURE<br><br>ACUTE EXACERBATION OF COPD<br><br>POST COVID SEQUELAE<br><br>K/C/O DM, HT,dry cough and breathlessness since 2 weeks.	Yes	No	No	24/10/21 : CXR : diffuse infilterates 60-70%, 16/25 HRCT	14	6	SEVERE COVID	<90%	
	CVD-44	32	Female?	HOSPITAL ACQUIRED INFECTIONS (VAP, CLABSI)<br>G3P2L2 WITH 11 WEEKS OF GESTATION S/P THREATEND ABORTION,C/o Fever since 16/6/21<br>Cough since 16/6/21<br>Shortness of breath since 16/6/21<br>Spotting PV with abdominal pain since 26/6/21	Yes	YEs	No	18/8/21 : CXR : B/L infilterates 30%	10	54	SEVERE COVID	<90%	
