# Supplementary material for: Molecular markers for early stratification of disease severity and progression in COVID-19
Source: Biol Methods Protoc. 2022 Nov 2;7(1):bpac028. doi: 10.1093/biomethods/bpac028 (PMC9731223; doi:10.1093/biomethods/bpac028)
Supplement: bpac028_Supplementary_Data [file bpac028_supplementary_data.zip › Supplementary Table 2_Octo2022.pdf]

Supplementary Table 2: IL6, IL8 and SPD levels of Cytokine-independent and Cytoline-dependent patients with their hospital course

Category	Research lab Serial no.	Age	Dexamethasone	Remedesivir	Tocilizumab	Chest X-ray (CXR)	Symptoms to Hospitalization (Day)	Hospital stay (Days)	IL6 (pg/ml)	IL8 (pg/ml)	SPD (ng/ml)	CRP (mg/dl)	d-Dimer (ng/ml)
Cytokine Dependent	Patient 14	21-25	Yes	Yes	No	CXR: B/L UPPER MID AND LOWER ZONE OPACITIES 60% INVOLVEMENT	4	14	521.21 ±7.33	643.94 ±1.48	148.70 ±2.21	28.35	620
	Patient 15	56-60	Yes	No	No	CT KUB (PLAIN) IMP:Bilateral bulky kidneys with perinephric and periureteric fat stranding, bilateral hydroureteronephro sis (R >L) with urothelial thickening - s/o Pyelonephritis.	7	25	662.07 ±6.09	305.96 ± 2.59	194.92 ±0.80	17.13	609
	Patient 22	61-65	Yes	Yes	No	EXTENSIVE SHADOWS 70-80% INVOLVEMENT	7	25	444.08 ± 8.25	643.42 ±1.23	130.89 ± 0.26	3.78	281
	Patient 28	66-70	Yes	No	No	Chest Xray-B/L patchy infilterates 70% lung involvement	1	6th day , Death	649.70 ±0.44	255.99 2±2.53	121.56 ±1.13	15.75	7088
	Patient 37	56-60	Yes	Yes	Yes	CXR : B/L infilterates 90-95% involvement	3	22nd Day death	638.40 ±33.52	276.59 ±0.65	138.84 ±0.25	21.89	556
	Patient 42	81-85	Yes	No	No	CXR : diffuse infilterates 60-70%, 16/25 HRCT	14	6	402.67 ± 18.21	275.65 ±5.90	147.79 ± 0.05	4.88	952
	Patient 44	31-35	Yes	Yes	No	CXR : B/L infilterates 30%	10	54 day, death	361.97 ± 18.21	57.50± 3.94	204.84 ± 1.46	4.10	6203
Cytokine independent	Patient 08	71-75	Yes	No	No	CXR: B/L NON HOMOGENOUS OPACITIES 40-50% INVOLVEMENT	10	10	248.98 ±3.66	592.23 ±2.72	13.67 ±0.15	10.97	6863
	Patient 16	66-70	Yes	Yes	No	CXR: 40% INVOLVEMENT MID AND LOWER ZONE INFILTRATES	7	17	209.25 ±4.19	36.74± 11.26	61.69 ±1.58	14.62	628
	Patient 20	31-35	Yes	No	No	EXTENSIVE GROUND GLASSING	5	Day6 shifted to ICU, Death	175.83 ±50.34	22.94± 0.01	27.86 ±1.11	4.50	196
	Patient-30	36-40	Yes	Yes	No	B/L costophrenic and left cardiophrenia angle blunting. Opacities on left lower and upper zone. Pleural effsuion minimal to moderate, left more than right. 60 - 70 % involvement	4	11	134.6 ±9.555	20.58 ±0.548	43.96 ±0.256	24.86	632
	Patient-33	36-40	Yes	?	?	8/7/21 : CXR : B/L diffuse infilterates with >90 % involvement, 26/26 HRCT	2	28th Death	224.19 ±1.17	108.18 ±5.47	31.12 ±1.53	23.67	758
	Patient-34	41-45	Yes	Yes	No	Steroid induced hyperglycemia,Cou gh since 4 days/ Dyspnea since 4 days	4	8	236.72 ±2.23	187.64 ±5.14	59.22 ±5.89	26.28	926
	Patient-36	61-65	Yes	No	No	17/6/21 : Right diffuse 25% infilterates 9/40	2	8	275.44 ±1.34	71.97 ±4.71	37.59 ±1.40	1.28	450
	Patient 39	61-65	Yes	Yes	No	CXR : B/L infilterates 80%	6	33	171.16 ±4.52	190.99 ±2.28	4.74 ±0.92	6.85	646
	Patient 40	76-80	Yes	Yes	No	CXR : B/L infilterates with 60 % involvement c. CT - HRCT-21/6/21- Partial/chronic pulmonary thrombo embolism involving the left lower lobar subsegmenta,l branches., 24/25, HRCT, >90% infiltrates Features of typical COVID Pneumonia - CORADS 6 with CT involvement score of 24 /25 (severe).	6	18	238.77 ±23.02	142.85 ±12.73	12.60 ±1.53	3.97	7003
